# Retrospective Analysis of Clinical and Radiologic Data Regarding Zygomatic Implant Rehabilitation with a Long-Term Follow-Up

**DOI:** 10.3390/ijerph182412963

**Published:** 2021-12-08

**Authors:** Michele Di Cosola, Andrea Ballini, Khrystyna Zhurakivska, Alberto Ceccarello, Riccardo Nocini, Annarita Malcangi, Giorgio Mori, Lorenzo Lo Muzio, Stefania Cantore, Antonio Olivo

**Affiliations:** 1 Department of Clinical and Experimental Medicine, Università degli Studi di Foggia, 71122 Foggia, Italy; michele.dicosola@unifg.it (M.D.C.); khrystyna.zhurakivska@unifg.it (K.Z.); giorgio.mori@unifg.it (G.M.); lorenzo.lomuzio@unifg.it (L.L.M.); 2 School of Medicine, University of Bari “Aldo Moro”, 70124 Bari, Italy; andrea.ballini@me.com; 3 Department of Precision Medicine, University of Campania “Luigi Vanvitelli”, 80138 Naples, Italy; 4 Faculty of Dentistry (Fakulteti i Mjekësisë Dentare-FMD), University of Medicine, 1001 Tirana, Albania; 5 Medical Center Padova, Private Practice, 3513 Padova, Italy; ceccarello.alberto@gmail.com (A.C.); a.olivo@medicalcenterpadova.it (A.O.); 6 Section of Ear Nose and Throat (ENT), Department of Surgical Sciences, Dentistry, Gynecology and Pediatric, University of Verona, 37126 Verona, Italy; riccardo.nocini@gmail.com; 7 Public Local Health Company (Azienda Sanitaria Locale, ASL), B.A.T, 76125 Trani, Italy; annarita.malcangi@gmail.com; 8 Department Interdisciplinary of Medicine, University of Bari “Aldo Moro”, 70124 Bari, Italy

**Keywords:** zygomatic implants, success rate, implant failure, oral and maxillo-facial surgery

## Abstract

Background: Zygomatic implants have been introduced to rehabilitate edentulous patients with severely atrophic maxillae. Their use has been reported by several studies, describing high overall survival rates at medium–long follow-up. The aim of this study was to retrospectively analyze if a few patient-related and implant-related features are correlated with implant success or the onset of complications. Materials and methods: Data of patients treated with zygomatic implants between May 2005 and November 2012 at three private clinics were collected and retrospectively analyzed. For each implant, the following data were collected: implant length, insertion path, ridge atrophy and sinus characteristics (width, pneumatization, thickness of mucosae, patency of sinus ostium). General patient characteristics and health status data were also recorded. The outcomes evaluated were implant failure, infective complications, early neurologic complications and overall complications. Results: A total of 33 patients (14 men, 17 women, mean age 59.1) that received 67 zygomatic implants were included in the study. The mean duration of the follow-up was of 141.6 months (min 109; max 198). In this period, a total of 16 (23.88%) implants in 8 (24.24%) patients were removed and 17 (51.51%) patients with 36 (53.73%) implants reported complications. Immediate loading resulted in a significantly lower risk of complications compared with the two-stage prosthetic rehabilitation (OR: 0.04, *p* = 0.002). A thickness of the sinus mucosa > 3 mm emerged to be correlated with a greater occurrence of infective complications (OR: 3.39, *p* = 0.019). Severe and extreme pneumatization of the sinus was significantly correlated with the incidence of overall complications (*p* = 0.037) and implant failure (*p* = 0.044). A large sinus width was predisposed to a higher risk of neurologic complications, infective complications and implant failure (*p* = 0.036, *p* = 0.032, *p* = 0.04, respectively). Conclusions: zygomatic implants are an alternative procedure for atrophic ridge rehabilitation when a conventional implant placement is not possible. Several clinical and anatomical factors can have a significant role in complication occurrence.

## 1. Introduction

Implant rehabilitation of edentulous patients is a widely used approach with well-standardized procedures and an excellent predictability [1]. The main limitation to the use of oral implants in several patients is represented by unfavorable anatomical features due to an extensive resorption or the degenerative processes of alveolar bones [2]. In such conditions, the procedure of implant placement and restoration may be a challenge. Several techniques of bone augmentation and the use of bone grafts have been proposed and implemented, making implant insertion and integration possible in compromised anatomical sites [2,3]. In addition, efforts have been made to find alternative extra-alveolar anchorage sites that permit implant rehabilitation in cases of extensive atrophy of the upper jaw [4,5].

Zygomatic implants (ZIs) were developed by Brånemark and proposed for the first time by Aparicio et al. in 1993 for the rehabilitation of seriously compromised maxillae [6]. In this approach, long screw-shaped implants were proposed to be anchored in the zygomatic bone, passing through the maxillary sinus [7]. Over the past three decades, several techniques and approaches have been described for ZI placement in order to improve performance and prosthetic rehabilitation [8,9]. In particular, special attention has been paid to the anatomy-guided approach for the implant insertion pathway [10].

Data about the complications and success of ZI rehabilitations vary significantly among studies. A recent systematic review summarized the available data, presenting a high overall survival rate (98.22%) with a minimum follow-up of 1 month and a maximum of 228 months reported in the included studies [10]. Nevertheless, only a few studies with long-term data are available in the literature [9,11,12,13]. In these studies, the survival rates vary between 94.32% and 100%. The most common reasons of failure were identified in missing integration and sinusitis that occurred immediately or during the follow-up period [12,13]. Furthermore, it is not clear if the choice of particular surgical or prosthetic techniques, rather than the intrinsic characteristics of the patients, may influence the implant failure. The purpose of this study was to analyze the long-term follow-up data of patients rehabilitated with ZIs in order to investigate if any correlation exists between the demographic, clinical, anatomical features, prosthetic timing or implant characteristics and the onset of complications or failures.

## 2. Materials and Methods

### 2.1. Study Design and Setting

The study was conducted and reported according to the STROBE recommendations [14]. This retrospective cross-sectional study investigated zygomatic implants placed in atrophic maxillae between May 2005 and November 2012 at the private clinics “Casa di Cura Diaz”, Padova (Italy), “Medical Center Padova”, Padova (Italy) and “Villa Pompea”, Bari (Italy) in collaboration with the University of Foggia by a team of three experienced surgeons and subjected to a follow-up evaluation in 2021. The length of experience of the surgeons was, on average, more than 50 zygomatic implants placed by each and more than 10 years of prosthetic experience.

The study was approved by the A.O.U. “Ospedali Riuniti” of Foggia in Foggia, Italy (N°65/CE/2021).

### 2.2. Participants

The participants were all patients treated with one or more ZI and a subsequent prosthetic rehabilitation of atrophic jaws. The fundamental requirement for the inclusion of patients in the study was the availability of preoperative (T0) computed tomography (CT).

### 2.3. Surgical Procedures and Postoperative Care

The study was carried out according to the Helsinki Declaration and informed written consent was obtained from all patients included in the study, who were informed about the benefits and risks associated with the procedure. All implant recipients were placed under general anesthesia and a local infiltration of 2% lidocaine with 1:100,000 adrenaline (Septodont) was performed. Antibiotic prophylaxis was made in all cases with Amoxicillin 3 g per day for 10 days. All patients were recommended rinses with 0.2% chlorhexidine and the use of a thermoplastic elastomeric (TPE) bristle during the first two weeks after the implant placement [10,14]. A full thickness flap was raised and the central/posterior part of the zygomatic complex was exposed, avoiding interference with the orbit. The flap elevation was performed in the way that access for the visualization of the infero-lateral aspect of the orbit was achieved to avoid trauma of the infraorbital nerve. The sinus membrane was carefully manipulated to move it away from the sinus walls into the sinus cavity. During the osteotomy preparation, saline-cooled irrigation was used to avoid overheating and rinse away debris. The osteotomy preparation was performed using the sequence of burs according to recommendations of the implant manufacturers. A maximum speed of 2000 rpm was reached during the preparation. Generally, the implant trajectory was planned from the posterior area of the maxillary ridge (premolar–molar region), passing through the sinus to reach the zygomatic bone. If two implants per side were planned, the anterior one was placed prior to the posterior with the emergence in the canine region. All implants were placed by hand. The prosthetic load, consisting of a provisional fixed acrylic prosthesis, was performed immediately in most cases (57/67, 85%). A final screw-retained prosthesis was delivered after 4–6 months. Only a few patients were subjected to a two-stage procedure because of a low implant stability quotient (ISQ < 60) with the first prosthetic load performed after 3 months of osseointegration. All clinicians followed the same surgical and prosthetic procedures.

All patients were placed in a follow-up protocol with a recommendation of periodic 6 month professional dental hygiene.

### 2.4. Implants

Two brands of zygomatic implants (Southern Implants, Irene, South Africa and Nobel Biocare, Goteborg, Sweden) were used. The implant length varied between 35 mm and 52.5 mm. The Nobel Biocare implants were characterized by a 45° head inclination and TiUnite surface. The Southern Implants had a 55° head inclination and were characterized by a Southern Implants Enhanced Surface.

### 2.5. Variables and Outcomes

The outcomes evaluated in this study were implant failure, infective complications and early neurologic complications. Furthermore, an outcome called “overall complications” including every type of complication that arose at any point in the follow-up (infective, hemorrhagic and neurologic) was created. The implant failure was assessed as any event resulting in a final loss of the implant during the follow-up period. The infective complications considered were sinusitis, an oroantral fistula and an infection of the soft tissues at any point after the implant placement. Early neurologic complications consisted of sensory deficits or motor nerve damage experienced by the patients in the first 2 months after surgery.

The implants were considered successful if, during the follow-up period, there was good stability and function as well as the absence of pain, infection and inflammation.

Data regarding sex, age, general health status, pathologies (diabetes, hypertension) and the smoking habits of the included patients were collected and summarized in an Excel sheet. Preoperative radiographies were examined using OnDemand3D™ Version 1.0.10.5385 software (Cybermed, Irvine, CA, USA), in which a multiplanar reconstruction may be viewed at intervals up to 0.5 mm. The following variables were defined and measured for each implant site:

*Implant path:* the implant insertion was decided based on the ZAGA classification [8]. The original ZAGA classes (0–4) were then dichotomized as completely intrasinusal (ZAGA 0) and not completely intrasinusal (ZAGA 1,2,3,4).

*Ridge atrophy:* the anatomy of the edentulous ridge was evaluated as described by Cawood and Howell [15] (II–V) and subsequently modified by Merli et al. [16] (II–V, A–C). Classes IIIc and IVc were then combined into one class for the statistical evaluation.

*Sinus width:* the sinus width was calculated on the sagittal sections as the distance between the medial and lateral walls of the sinus at a fixed vertical point (moving 15 mm from the lower edge of the alveolar ridge in a cranial direction). The obtained values were then distinguished into three classes, as suggested by Chan et al. [17]: narrow (<14 mm), medium (14–17 mm) and large (>17 mm).

*Sinus pneumatization:* the sinus pneumatization was measured as described by Tolstunov and colleagues [18] in 2012 and 5 classes were obtained (SP0, SP1, SP2, SP3 and SP4), which were subsequently merged into two groups: A (SP0, SP1 and SP2) and B (SP3 and SP4).

*Thickness of the sinus mucosa:* the thickness of the sinus mucosa was evaluated using a digital ruler according to Lana et al. [19] and classified into two groups: ≤3 mm and >3 mm. The cut-off of 3 mm was chosen in order to discriminate the physiologic or para-physiologic conditions from the pathologic thickness of the mucosa in case of sinusitis or asthma [20].

*The patency of sinus ostium* was also assessed.

*The deviation of nasal septum* was measured in degrees, as described in Janovic et al. in their article [21], and left as a continuous variable.

Finally, *the length of the implants* was divided into two categories: < 45 mm and ≥ 45 mm.

### 2.6. Statistical Analysis

For all the included variables, descriptive statistics were performed by calculating the mean and standard deviation for the continuous variables and the frequency percentages for the categorical variables. In order to investigate the associations between the complications (i.e., implant failure, infective and early neurologics as well as overall) and the investigated variables (i.e., implant path, ridge atrophy, sinus width, sinus pneumatization, thickness of the sinus mucosa, patency of the sinus ostium, deviation of the nasal septum and the length of the implant), multiple univariate logistic regression models were built. Furthermore, a stepwise multivariate logistic regression analysis was performed. All the analyses were performed with Stata 16.0 software (StataCorp LLC, 4905, College Station, TX, 77,845, USA) using a *p*-value lower than 0.05 as a threshold of statistical significance.

## 3. Results

### 3.1. Study Population

A total of 33 patients that received 67 zygomatic implants were included in the study. In particular, 3 patients received 4 implants (2 per side), 5 patients received 1 monolateral implant and 25 patients were rehabilitated with 2 zygomatic implants (1 per side). No patient suffered from chronic or acute sinusitis at the moment of surgery. The descriptive data of the included population are summarized in Table 1.

The main clinical and radiographic characteristics of the cohort used for the variable definitions are reported in Table 2. The mean duration of the follow-up was of 141.6 months with a minimum of 109 and a maximum of 198 months.

### 3.2. Outcomes

In this period, a total of 16 (23.88%) implants in 8 (24.24%) patients were removed. The reasons for failure were represented by the occurrence of oroantral communication and subsequent recurrent sinusitis that were fixed only after the implant removal. A total of 17 (51.51%) patients with 36 (53.73%) implants reported complications. In particular, 28 (41.79%) implants were affected by infective complications and 5 (15.15%) patients referred to early neurologic complications after the implant placement. The infective complications consisted of sinusitis in correspondence with 22 inserted implants; 6 implants showed soft tissue dehiscence. A summary of the outcomes is reported in Table 3.

### 3.3. Main Results

From the univariate logistic regression analysis, the following statistically significant correlations emerged. The timing of the prosthetic rehabilitation was revealed to be correlated with the onset of complications. In particular, an immediate loading resulted in a significantly lower risk of infective, neurological and overall complications compared with the two-step rehabilitation (OR: 0.17; 0.06 and 0.04, respectively). A thickness of the sinus mucosa > 3 mm emerged to be correlated with a greater occurrence of infective complications (*p* = 0.019).

Sinus pneumatization of type SP3 and SP4, corresponding with severe and extreme enlargements of the sinus, were significantly correlated with the incidence of overall complications (*p* = 0.037) and implant failure (*p* = 0.044). In other words, the presence of an extended sinus pneumatization exposed patients to a greater risk of complications (OR = 1.89) and implant failure (OR = 3.65) compared with the group of patients with a mild (SP1) or moderate (SP2) degree of pneumatization.

Regarding the demographic features, a logistic regression revealed a significant correlation (*p* < 0.05) between implant failure and gender, resulting in a higher risk of failure in males compared with females. No other evaluated conditions (i.e., age, smoking, hypertension, diabetes) were correlated with any of the considered outcomes.

The results of the univariate logistic regression analysis investigating the association between the clinical variables and the outcomes are summarized in Table 4 and Table 5. The results of the stepwise multivariate logistic regression confirmed the correlations of the univariate analysis. Furthermore, a significant positive correlation emerged between a large sinus width and the occurrence of overall complications (*p* = 0.004) including both neurologic (*p* = 0.036) and infective (*p* = 0.032) complications. The statistically significant correlations that emerged from the multivariate analysis are summarized in Table 6.

## 4. Discussion

Dental implants are widely used to replace missing teeth and rehabilitate edentulous patients. Immediately after the implant placement, the local blood vessel growth allows the recruitment of migratory mesenchymal stem cells to the surgical site and the implant surface. These cells then proliferate and differentiate into mature osteoblasts responsible for bone matrix formation, which is essential for implant integration [22,23]. However, in a few cases, the anatomy of the residual alveolar bone is unfavorable, precluding a sufficient anchorage of the implants. In these cases, procedures of bone graft may be needed, making surgical procedures more complex and prolonging the healing timing and postponing the final prosthetic rehabilitation. The implant survival rate in cases treated with a ridge augmentation has been reported to range from 75.57 to 100%, as summarized by a recent systematic review [1]. An alternative was proposed by Brånemark, who suggested the engagement of the zygomatic bone for implant anchorage in patients with extensively resorbed maxilla [7,24]. The intervention requires good surgical skills in order to properly place the implants and avoid damage to the surrounding anatomic structures. Nevertheless, the advantages are that in many cases bone augmentation is not required and prosthetic rehabilitation can be fitted immediately. Even if there are no published or ongoing randomized controlled clinical trials (RCTs) or even controlled clinical trials (CCTs) comparing the efficacy of zygomatic implants with various bone augmentation procedures for conventional implant placement, the data on the survival rates of zygomatic implants are encouraging [25]. A recent systematic review including data of 4556 ZIs with a follow-up of 12 years found a cumulative survival rate of 95.21% [26]. Another review with a shorter average follow-up reported a success rate of 98.22% [10]. The data of our study referred to an average follow-up of 12 years and the survival rate of the placed implants was around 76% (51/67), being significantly lower compared with those published by the majority of the studies. However, similar rates were reported by Rodríguez-Chessa (79.1%) [27] and Yates (86%) [28].

The mean age of our patients was of 59 years, similar to those generally reported in the literature and the distribution between the sexes was quite equal. The length of placed implants varied between 35 and 52.5 mm and the implants presented three types of surface (rough-anodized, machined and SIES surfaces). Neither the implant brand nor the other characteristics resulted in significantly influencing the implant success.

The most frequent complications reported in the literature are sinusitis followed by gingival infection around the implants and oroantral fistulas, so the use of mouthwash or a soft bristle in the follow-up is recommended. Della Nave et al. [29] described a global incidence of complications of about 4.5% in a mean follow-up period of 3.16 years. Other studies mention an incidence of complications that varies between 0 and 26% [30,31] However, Chrcanovic and collaborators, in their systematic review on survival and complications of ZIs, state that the data reported in the available studies are not standardized so they are difficult to compare [26]. In our study, the incidence of complications was separately investigated for infective, early term neurologic and overall complications. Regarding the infective complications, all types of infections were considered, grouping together the early, mild and long-term complications. The rate of infective complications in our patients was relatively high, occurring in 41.79% of the placed implants. However, this data should be interpreted considering an all-encompassing approach and the long follow-up adopted in this study. Regarding the potential influence of clinical or general factors on the success rate and the onset of complications, few significant correlations were identified. In particular, none of the general characteristics (smoking, hypertension, diabetes) were correlated with failure or complications. Conversely, the timing of the prosthetic rehabilitation significantly correlated with the incidence of complications with the immediate loaded prosthesis associated with a lower occurrence of infective, neurologic and overall complications (*p* < 0.05) compared with the delayed prosthetic load. Considering that the implants with a two-stage prosthetic rehabilitation were few in this study (10/67), the statistical power of this result was not high. An explanation can be found in the fact that the implants loaded in the two-stage procedure were at a high risk from the beginning as shown by the low ISQ [32]. Nevertheless, the same conclusion was reached in a recent review where the difference of the ZI survival rates between the immediate and delayed protocols were shown to be statistically significant (*p* = 0.003), resulting in an immediate load to give higher survival rates [26]. Further studies on this topic are required as, in the most part of the available studies, only one of the two approaches is usually reported without comparative results.

Another factor that resulted in a significant increase of infective complications was a thickness of the sinus mucosae greater than 3 mm (OR: 3.39, *p* = 0.019). The median thickness of the Schneiderian membrane reported in the literature is 1.03 mm and several authors have stated that the insertion of ZIs leads to an increase in the membrane thickness over time [33]. This phenomenon is explained by several reactive and inflammatory processes that occur after the implant placement. No data are available about the correlation between the mucosa thickness and implant success. Nevertheless, an initial condition of an enlarged sinus mucosa can be a sign of a chronic inflammation or infection and may be predisposed to a subsequent exacerbation after the implant placement [33].

The factors that were correlated with an implant failure were the degree of sinus pneumatization (*p* = 0.044) and prosthetic timing. Large sinuses seemed to predispose the patients to a higher risk of implant failure and occurrence of infective and neurologic complications. The measurements of sinus pneumatization were performed following criteria described by Tolstunov et al. [18]. According to methods described by the authors, five categories of sinus size were defined: SP1 (mild degree of pneumatization), SP2 (moderate degree of enlargement), SP3 and SP4 (severe and extreme degree of enlargement, respectively) and SP0 (clear and high/small sinus, not interfering with implant treatment). For statistical purposes, the single categories were subsequently merged in two groups in our study: A (SP0, SP1 and SP2) and B (SP3 and SP4). The included cohort was equally distributed between two groups (47.76% and 52.23%, respectively, for A and B). These data confirmed that the population included in our study had a considerable enlargement of the maxillary sinus compared with the data of the general edentulous population reported by Tolstunov et al. [18], where the greatest proportion (78.3%) of patients was characterized by clear, mild or moderate sinus pneumatization with a sufficient amount of maxillary bone to allow a full arch traditional implant treatment. As suggested by the authors, patients of the SP3 category may require an additional bone graft/sinus lift whereas in SP4 patients, an implant treatment may not be possible, making large bone grafting or zygoma implants necessary. In our sample, extensive sinus enlargement was associated with a greater incidence of implant failure (*p* = 0.044) and the occurrence of complications (*p* = 0.037) compared with mild or moderate sinus pneumatization. 

The limitations of the study are represented by a relatively small sample size and its retrospective design. Moreover, as the interventions were performed several years before the publication of this study, several procedures (i.e., the antibiotic protocol and radiographic setup) are not updated to nowadays standards. Nevertheless, the long follow-up of the study offers important data that can integrate with the available scientific scenario.

## 5. Conclusions

The use of zygomatic implants is an alternative procedure, making implant rehabilitation possible of extensively atrophic maxilla when conventional procedures are not possible. Several anatomical and procedural factors such as sinus conformation and the thickness of the sinus mucosa may influence the overall success of the zygomatic implant rehabilitation and be predisposed to a higher occurrence of complications. Further studies are necessary to investigate and confirm these correlations.

## Figures and Tables

**Table 1:** Descriptive data of the participants.

	Patients (*n*)	Implants (*n*)	Mean Age at Enrolment (Years ± sd)	Hypertension (Yes:No)	Diabetes (Yes:No)	Smoking (Yes:No)	Follow-Up (Months ± sd)	Failures (*n* Patients (*n* Implants))
**Total**	33	67	59.1 ± 8.68	6:27	5:28	7:26	141.6 ± 25.34	8 (16)
**Sex (%)** **Male**	14	28	60.2 ± 9.26	3:11	3:11	6:8	136.92 ± 26.48	5 (10)
**Female**	19	39	58.4 ± 8.41	3:16	2:17	1:18	145.05 ± 24.62	3 (6)

*n*: number; SD: standard deviation.

**Table 2:** Implant level characteristics of the cohort.

Implant Level Variables	*n* = 67 *n* %	
**Implant path**		
Completely intrasinusal	47	70.15
Not completely intrasinusal	20	29.85
**Ridge atrophy**		
*IIc*	26	38
*IIIc–IVc*	21	31.34
*Vc*	20	29.85
**Sinus width**		
Narrow	12	17.91
Medium	16	23.88
Large	39	58.20
**Sinus pneumatization**		
A	32	47.76
B	35	52.23
**Thickness of the sinus mucosa**		
≤3 mm	40	59.7
>3 mm	27	40.3
**Patency of sinus ostium**		
Yes	49	73.13
No	18	26.86
**Implants** **length**		
<45 mm	44	65.67
≥45 mm	23	34.33
**Implant brand**		
Nobel Biocare	45	67.16
Southern Implants	22	32.83
**Prosthetic timing**		
Immediate loading	57	85.07
Deferred loading	10	14.92
**Septum deviation**	Average 12.11 ± 7.78	

*n*: number; %: percentage of the total.

**Table 3:** Data on the failure and complications at the implant and patient levels.

Outcome	Implants (*n* = 67)	Patients (*n* = 33)
Failure	16 (23.88%)	8 (24.24%)
Overall complications	36 (53.73%)	17 (51.51%)
Infective complications	28 (41.79%)	15 (45.45%)
Early neurological complications	11 (16.41%)	5 (15.15%)

*n*: number; % percentage of the total.

**Table 4:** Univariate logistic regression model to investigate the association of clinical variables with infective complications and early neurologic complications.

Variables		Infective Complications		Early Neurologic	
		OR 95% (CI)	*p*-Value	OR 95% (CI)	*p*-Value
**Implant** **path**	Completely intrasinusal (ref)	1.00	-	1.00	-
	Not completely intrasinusal	0.49 (0.16−1.48)	0.206	0.86 (0.20−3.64)	0.838
**Prosthetic** **timing**	Deferred loading (ref)	1.00	-	1.00	-
	Immediate loading	0.17 (0.04−0.72)	0.016 *	0.06 (0.012−0.26)	0.001 *
**Implant** **length**	<45 mm (ref)	1.00	-	1.00	-
	≥45 mm	1.06 (0.37−3.03)	0.905	0.18 (0.02−1.51)	0.114
**Implant** **brand**	Nobel (ref)	1.00	-	1.00	-
	SI	1.07 (0.40−2.88)	0.881	0.26 (0.05−1.29)	0.100
**Sinus** **pneumatization**	A (ref)	1.00	-	1.00	-
	B	2.33 (0.86−6.33)	0.097	2.86 (0.69−11.93)	0.148
**Ridge** **atrophy**	IIc (ref)	1.00	-	1.00	-
	IIIc–IVc	0.68 (0.21−2.25)	0.530	0.58 (0.09−3.51)	0.553
	Vc	1.36 (0.42−4.40)	0.604	1.83 (0.42−7.97)	0.604
**Sinus** **width**	Narrow (ref)	1.00	-	1.00	-
	Medium	0.69 (0.11−4.24)	0.691	1.15 (0.16−8.27)	0.887
	Large	3.88 (0.91−16.6)	0.067	0.91 (0.16−5.23)	0.915
**Thickness of** **sinus mucosa**	≤3 (ref)	1.00	-	1.00	-
	>3	3.39 (1.22−9.44)	0.019 *	1.29 (0.35−4.74)	0.703
**Smoking**	No (ref)	1.00	-	1.00	-
	Yes	2.72 (0.78−9.46)	0.116	0.42 (0.13−1.44)	0.314
**Ostium** **patency**	No (ref)	1.00	-	1.00	-
	Yes	2.25 (0.22−22.8)	0.493	1.34 (0.47−2.11)	0.665
**Septum** **Deviation**		1.01(0.97−1.07)	0.521	1.03 (0.96−1.10)	0.447

OR: odds ratio; CI: 95% confidence interval; ref: reference; * statistically significant (*p* < 0.05).

**Table 5:** Univariate logistic regression model to investigate the association of clinical variables with implant failure and overall complications.

Variables		Implant Failure		Overall Complications	
		OR 95% (CI)	*p*-Value	OR 95% (CI)	*p*-Value
**Implant** **path**	Completely intrasinusal (ref)	1.00	-	1.00	-
	Not completely intrasinusal	0.73 (0.20−2.61)	0.628	0.81 (0.28−2.3)	0.690
**Prosthetic** **timing**	Deferred loading (ref)	1.00	-	1.00	
	Immediate loading	0.35 (0.09−1.31)	0.120	0.04 (0.004−0.29)	0.002 *
**Implant** **length**	<45 mm (ref)	1.00	-	1.00	-
	≥45 mm	2.92 (0.91−9.37)	0.071	0.92 (0.33−2.60)	0.881
**Implant** **brand**	Nobel(ref)	1.00	-	1.00	-
	SI	0.55 (0.17−1.82)	0.331	0.61 (0.22−1.69)	0.345
**Sinus** **pneumatization**	A (ref)	1.00	-	1.00	-
	B	3.65 (1.04−12.86)	0.044 *	1.89 (1.04−3.46)	0.037 *
**Ridge** **atrophy**	IIc (ref)	1.00	-	1.00	-
	IIIc–IVc	1.31 (0.32−5.32)	0.703	0.64 (0.19−2.20)	0.478
	Vc	1.8 (0.46−7.05)	0.399	1.6 (0.49−5.21)	0.435
**Sinus** **width**	Narrow (ref)		1.00	-	1.00
	Medium	0.2 (0.02−2.22)	0.190	0.46 (0.08−2.62)	0.383
	Large	1.33 (0.30−5.81)	0.702	1.9 (0.49−7.36)	0.353
**Thickness of** **sinus mucosa**	≤3 (ref)	1.00	-	1.00	-
	>3	1.68 (0.54−5.22)	0.367	2.51 (0.91−6.92)	0.075
**Smoking**	No (ref)	1.00	-	1.00	-
	Yes	2.44 (0.67−8.95)	0.178	0.40 (0.10−1.64)	0.204
**Ostium** **patency**	No (ref)	1.00	-	1.00	-
	Yes	0.94 (0.09−9.69)	0.957	1.97 (0.19−20.06)	0.565
**Septum** **deviation**		0.97 (0.91−1.03)	0.274	1.01 (0.95−1.05)	0.922

OR: odds ratio; CI: 95% confidence interval; ref: reference; * statistically significant (*p* < 0.05).

**Table 6:** Multivariate stepwise logistic regression model investigating the association of the independent variables with the evaluated outcomes.

Variables		Infective Complications		Early Neurologic Complications		Overall Complications		Implant Failure	
		OR 95 % (CI)	*p*-Value	OR 95 % (CI)	*p*-Value	OR 95 % (CI)	*p*-Value	OR 95 % (CI)	*p*-Value
Prosthetic Timing	Deferred loading (ref) Immediate loading	1.00 0.115 (0.02–0.54)	0.007 *	1.00 0.001 (0.00–0.20)	0.013 *				
Sinus Width	Narrow (ref) Medium Large	1.00 1.515 (0.18–12.44) 6.400 (1.17–34.79)	0.699 0.032 *	1.00 1.01 (0.39–12.80) 2.421 (1.86–22.27)	0.233 0.036 *	1.00 4.133 (0.59–49.07) 9.320 (1.66–67.10)	0.051 0.004 *		
Thickness Of Sinus Mucosa	≤3 (ref) >3	1.00 2.758 (0.94–8.03)	0.045 *						
Sinus Pneumatization	A (ref) B					1.00 3.023 (1.04–9.46)	0.045 *	1.00 2.60 (1.21–5.62)	0.014 *

CI: 95% confidence interval; ref: reference; * statistically significant (*p* < 0.05).

## Data Availability

Data are contained within the article.
